# Comorbidities, health-related quality of life and its associated factors among people living with HIV/AIDS in Gandaki Province of Nepal

**DOI:** 10.1186/s12889-026-28192-5

**Published:** 2026-06-20

**Authors:** Srijana Paudel, Sushila Baral, Rajesh Kumar Yadav, Yadu Nath Baral, Dipendra Kumar Yadav, Santosh Poudel, Khim Bahadur Khadka, Amar Nagila, Bipin Adhikari

**Affiliations:** 1 Pokhara Academy of Health Sciences, Pokhara, Nepal; 2 School of Health and Allied Sciences, Pokhara University, Pokhara, Nepal; 3 Global Fund, Save the Children International, Kathmandu, Nepal; 4 Islington college, London Metropolitan University Degree, Kathmandu, Nepal; 5 Health Directorate, Pokhara, Gandaki Province Nepal; 6 Mahidol-Oxford Tropical Medicine Research Unit, Faculty of Tropical Medicine, Mahidol University, Bangkok, Thailand; 7 Center for Tropical Medicine and Global Health, Nuffield Department of Clinical Medicine, University of Oxford, Oxford, UK

**Keywords:** Comorbidities, Health-related quality of life, HIV/AIDS, Nepal

## Abstract

**Background:**

HIV/AIDS remains a public health concern globally. Comorbidities add to the disease burden among people living with HIV (PLHIV) and may be associated with poorer health-related quality of life. This study aimed to assess the prevalence of comorbidities, health-related quality of life (HRQoL), and associated factors among people living with HIV in Gandaki Province, Nepal.

**Methods:**

A cross-sectional study was conducted at the antiretroviral therapy (ART) centre of Western Regional Hospital, Pokhara. PLHIV registered at the ART centre were selected using simple random sampling. A multivariable logistic regression model was used to explore factors associated with HRQoL. Adjusted odds ratios (AORs) with 95% confidence intervals (CIs) were reported.

**Results:**

A total of 337 PLHIV participated in the study. The mean overall HRQoL score was 6.25 ± 1.87, and 52.2% of participants reported poor quality of life. At least one comorbidity was present in 28.2% of participants. The most frequently reported comorbidities were hypertension (36.8%), diabetes (33.7%), tuberculosis (14.9%), hepatitis B (10.6%), and hepatitis C (4.3%). PLHIV with comorbidities had higher odds of poor HRQoL compared with those without comorbidities (AOR = 1.65; 95% CI: 0.98–2.78). Factors associated with comorbidities included marital status, family structure, education, occupation, current alcohol consumption, having a migrant worker spouse, CD4 count, transportation costs, perceived behaviour of healthcare workers, and HRQoL across all except the physical domain.

**Conclusions:**

Comorbidities were common among people living with HIV. The findings suggest a high prevalence of poor quality of life among PLHIV, with comorbidities potentially associated with poorer HRQoL. These findings underscore the need for integrated interventions that address both health and social factors, including community engagement strategies.

**Supplementary Information:**

The online version contains supplementary material available at https://doi.org/10.1186/s12889-026-28192-5.

## Background

Human immunodeficiency virus (HIV) continues to be a serious public health problem affecting 40.1 million people globally [1]. According to the World Health Organization (WHO) in 2021, 650, 000 people died from HIV related causes and 1.5 million people acquired HIV [2]. With increasing access to effective HIV prevention, diagnosis, treatment and care, including for opportunistic infections, HIV infection has become a manageable chronic health condition, enabling people living with HIV to lead long and healthy lives [2–4]. Globally, 31 million people living with HIV were receiving ART in 2023 [5]. The continuous implementation of combined antiretroviral therapy in clinical practice since its introduction has substantially improved the clinical outcome of people living with HIV (PLHIV), transforming HIV infection into a chronic disease and increasing patients’ life expectancy. ART not only prolongs the lives of PLHIV but also improves their quality of life (QoL), allowing them to return to work, take care of their families, and contribute to their communities similarly to those without HIV [6–8].

Studies have shown that the most common comorbidities among people living with HIV include diabetes mellitus, cardiovascular diseases, and respiratory and hepatic diseases. Renal diseases, substance abuse and dependence, sexually transmitted infections (herpes simplex, syphilis, gonorrhoea and *Mycoplasma genitalium*) and psychiatric disorders (including depression, anxiety, schizophrenia and cognitive impairment) are also more prevalent among PLHIV [9]. A study from Nepal reported that the prevalence of hypertension and diabetes among PLHIV was 19.6% and 9.9%, respectively [10, 11].

Although the quality of life of PLHIV has improved with treatment, the chronic nature of the conditions may still affect HRQoL scores [12]. PLHIV continue to contribute substantially to the global disease burden [13].

Between 1990 and 2015, 34 million people died from AIDS-related causes, with individuals aged 15–24 years accounting for about one-third of new HIV infections globally. In 2019, 38 million people were living with HIV and 1.7 million people were newly diagnosed. AIDS-related illnesses were responsible for 690,000 deaths. Among the newly diagnosed HIV infections, about 61% were in sub–Saharan Africa. For instance, in Ethiopia, 7194 people were newly diagnosed in 2018 which had increased to 8257 in 2022 [14, 15].

Quality of life is a broad multidimensional concept that usually includes subjective evaluations of both positive and negative aspects of life; and has been recognized as a prominent measurement tool in epidemiological studies and clinical trials including among people living with HIV and the AIDS [16]. When quality of life is considered in the context of health and diseases, it is commonly referred to as HRQoL to differentiate it from other aspects of quality of life [17–19]. HRQoL refers to how well a person functions in their life, and their perceived wellbeing in the physical, mental and social domains of health [10–13].

The HRQoL of PLHIV is an important indicator of the effectiveness of HIV/AIDS treatment and care programmes, as reported by the patients [19]. Such assessments can also be useful in identifying factors associated with HRQoL among PLHIV which may help inform public health interventions aimed at improving HRQoL [14, 20].

ART care service can use HRQoL measures to monitor patients’ needs, improve engagement and retention in care. Studies across the world have shown that residence, educational status, social support, employment status, duration of treatment, drug side effect, opportunistic infection, CD4 + count, WHO staging, viral load, sex, and marital status are associated with HR-QoL in individuals living with HIV on ART [8, 21–24]. A meta-analysis of studies conducted in Ethiopia found poor HRQoL among PLHIV with comorbidities. However, in the context of Nepal, the prevalence of comorbidities and factors associated with health-related quality of life among PLHIV remains underexplored. The main objective of this study was to assess the prevalence of comorbidities, HRQoL, and associated factors among PLHIV in Gandaki province in Western Nepal.

## Methods

### Study area and study design

A cross-sectional study was conducted among 337 PLHIV registered at ART center of Western Regional Hospital (WRH) in Gandaki province, Nepal from 1 April to 15 July 2023. The hospital is located in the capital city of Gandaki province.

### Study participants

The study population comprised people living with HIV (PLHIV) registered at the ART center of WRH, who had completed at least 60 days of antiretroviral therapy. PLHIV aged 18 years and above were considered eligible for inclusion. Participants were informed about the study objectives, and a written informed consent was obtained before data collection. A random sampling technique was used. A tertiary care hospital with the highest PLHIV patient flow was selected for the study. A sampling frame was obtained from the selected hospital, where 1326 PLHIV were enrolled in ART services. Using the Cochrane sample size formula with a proportion (p) of 0.46 [8] and a margin of error (d) of 0.05, the calculated sample size was 337. Microsoft Excel spreadsheet was used to generate random numbers, and samples were selected accordingly. The list of patients was obtained from the hospital with assurances of confidentiality. Participants with severe mental illness who were unable to respond to our questions were excluded.

### Variables and questionnaire

Various factors were considered in the study as dependent (HRQoL) and independent variables. An extensive literature review and consultations with experts were conducted in addition to pretesting the questionnaire on 10% of the sample. The factors were broadly categorized into socio demographic, disease-related, lifestyle-related and healthcare system-related variables. These factors were examined to better understand their associations within the context of the study.

### Data collection and statistical analysis

Data were collected from the PLHIV using an interviewer-administered questionnaire in Nepali. The questionnaire was divided into three sections. The first section included socio-demographic characteristics and disease-related information. The second section focused on comorbidities, associated factors, and lifestyle-related behaviours of participants. The third section assessed HRQoL using the Nepali version of the World Health Organization Quality of Life-HIV BREF (WHOQoL-BREF-HIV).

The WHOQoL-BREF-HIV is a 31-item instrument developed by the World Health Organization to assess HRQoL among PLHIV. It is a shortened adaptation of the WHOQoL-HIV, retaining core domains including physical health, psychological well-being, social relationships, environment, spirituality and level of independence (Supplementary File 1). Each item is rated on a 5-point Likert scale, with higher mean scores indicating better quality of life in that domain. Overall quality of life was determined by computing domain scores, with values greater than 50% representing good quality of life and those ≤ 50% denoting poor quality of life [25].

The tool has been widely validated in Nepal and provides a reliable measure of overall and domain-specific quality of life. Regarding comorbidities, the questionnaire included the ten common diseases, and respondents reporting at least one of these conditions were considered to have a comorbidity.

Data were collected by face-to-face interviews with the help of the interview questionnaire. Respondents were selected randomly from the sampling frame derived from the list provided by the hospital. Participants’ responses were recorded in the questionnaire. Data were entered into EpiData software (version 3.1), and analysis was performed using the Statistical Package for Social Science (SPSS, version 16). Univariable analysis was computed to describe socio-demographic profile of participants, the prevalence of comorbidities and level of Health-related quality of life, while mean and standard deviation were calculated for continuous variables. Pretesting was conducted among 10% of the sample size.

Bivariable logistic regression, Chi-squared, and Fisher’s exact tests were used to assess factors associated with comorbidities among PLHIV. In multivariable analysis, variables that were statistically significant in bivariable analysis were included. A multivariable logistic regression model was used to identify independent factors associated with HRQoL and comorbidities. Mean values were used to determine the level of quality of life. Association between comorbidities, HRQoL and Independent Variables were assessed using logistic regression, reporting odds ratios (ORs) with 95% confidence intervals (CIs). The Hosmer–Lemeshow goodness-of-fit test was used to assess the fit of the logistic regression model. Potential interactions between key variables were also examined. The results were considered significant at *p* < 0.05.

## Results

### Participant characteristics

A total of 337 people living with HIV(PLHIV) participated in the study. Majority of the participants (70.9%) were above 35 years old, and 50.4% were female. Most respondents (80.1%) in the study were identified as Hindu. Approximately 79.8% of individuals were enrolled in a health insurance scheme (Table 1).

**Table 1:** Socio-demographic characteristics of the participants

Characteristics	Frequency	Percentage
Age		
< 25 Years	41	12.2
25–35 Years	57	16.9
> 35 Years	239	70.9
Gender		
Male	167	49.6
Female	170	50.4
Religion		
Hinduism	270	80.1
Buddhism	56	16.6
Christianity	10	3.0
Islam	1	0.3
Ethnicity		
Dalit	83	24.6
Disadvantaged non-dalit Terai caste	7	2.1
Disadvantaged Janjati	31	9.2
Religious minorities	6	1.8
Upper caste	86	25.5
Relatively advantaged Janajati	124	36.4
Marital Status		
Single	49	14.5
Married	230	68.2
Divorced	9	2.7
Widow/ Widower	45	13.4
Separated	4	1.2
Family Type		
Nuclear	287	85.2
Joint	50	14.8
Educational status		
Illiterate	39	11.6
Non-formal education	41	12.2
Basic education (1–8 Class)	145	43.0
Secondary education (9–12 Class)	99	29.4
Higher education (completion of bachelor or above)	13	3.9
Enrolment in health insurance scheme		
No	68	20.2
Yes	269	79.8

### Prevalence and types of comorbidities

Overall, 28.2% of participants reported having at least one comorbidity. The most commonly reported conditions were hypertension (36.2%), and diabetes (31.9%), followed by tuberculosis (14.9%). Co-infection with hepatitis B was seen in minority of study population (10.6%) while hepatitis C in very few of respondents (4.3%) (Table 2).

**Table 2:** Prevalence of comorbidities and types of comorbidities among Human Immunodeficiency Virus Patients

Characteristics	Frequency	Percentage
Prevalence of comorbidities		
No	245	71.8
Yes	95	28.2
Types of co-morbidities**(multiple response)		
Hypertension	35	36.8
Diabetes mellitus	32	33.7
Tuberculosis	14	14.9
Hepatitis B	10	10.6
Cardio-vascular Disease	7	7.4
Cancer	6	6.3
Asthma	6	6.3
Thyroid	6	6.3
Hepatitis C	4	4.3
Health related Quality of Life		
Good	161	47.8
Poor	176	52.2

### Health Related Quality of Life (HRQoL)

More than half of the PLHIV respondents (52.2%) were classified as having poor overall HRQoL. Participants with comorbidities had lower mean scores across several domains, including physical health (10.94**±**2.20), psychological health (15.16**±**2.50), level of independence (10.45**±**2.79), and social relations (11.27**±**2.63) (Table 3).

**Table 3:** Association between non-comorbidities and comorbidities among PLHIV

HRQOL domain	Non-comorbidities (mean±SD)	Comorbidities (mean±SD)	*P*-value
Physical health	11.26±2.11	10.94±2.20	0.212
Psychological health	16.16±2.08	15.16±2.50	< 0.001**
Level of independence	11.74±1.87	10.45±2.79	< 0.001**
Social relations	12.39±2.22	11.27±2.63	< 0.001**
Environmental health	25.69±3.68	24.07±4.72	< 0.001**
Spirituality belief	10.14±2.19	10.91±2.33	< 0.005**
Overall Quality of Life	6.92±1.36	4.71±1.94	< 0.001**

*HRQOL* Health Related Quality of Life

** Statistically highly significant. independent sample T test

### Factors associated with HRQoL

Table 4 shows adjusted relationship of explanatory variables with quality of life. Age, ethnicity, ART enrollment, and health worker’s behaviors were not found statistically significant whereas, co-morbidities, time favorable (10:00 am-12:00 am) were found to be associated with HRQoL. Participants without comorbidities had higher odds of reporting better HRQoL compared to those without comorbidities (aOR = 1.647, 95% CI: 0.98–2.78, *p* = 0.042).

**Table 4:** Adjusted relationship of explanatory variables with HRQoL

Characteristics	COR	aOR	95% CI	*P*-value
Age				
< 25 years	1.215	1.167	0.580–2.348	0.091
25–35 years	2.188	1.988	1.075–3.676	
≥35 years	**1**	**1**	**Ref**	
Ethnicity				
Dalit	2.020	2.007	1.135–3.549	0.056
Upper caste group	1.106	1.218	0.704–2.105	
Relatively advantaged janajati and others caste	**1**	**1**	**Ref**	
Antiretroviral Therapy enrollment				
< 5 years	1.199	1.316	0.831–2.083	0.242
> 5 years	**1**	**1**	**Ref**	
Comorbidities				
No	1.862	1.647	0.977–2.775	0.042*
Yes	**1**	**1**	**Ref**	
Time favorable				
No	**1**	**1**	**Ref**	0.049*
Yes	1.573	1.742	1.012–2.997	
Health workers behave				
Very friendly	1.421	1.638	0.640–4.192	0.050
Friendly	0.964	0.917	0.426–1.976	
Indifferent	0.345	0.415	0.146–1.179	
Unfriendly	**1**	**1**	**Ref**	

* Statistically significant; References are in bold

### Factors associated with comorbidities

Bivariable analysis indicated that CD4 count and perceived behaviour of healthcare workers were associated with the presence of comorbidities. As illustrated in Table 5, participants reporting very friendly behaviour from healthcare workers had higher odds of reporting comorbid conditions (OR = 1.67; 95% CI: 0.55–5.09; *p* = 0.007). Similarly, CD4 count showed a statistically significant association with comorbidity status (*p* = 0.004).

**Table 5:** Adjusted relationship of explanatory variables with comorbidities

Characteristics	COR	aOR	95%,CI	*P* value
Marital status				
Single	1	**1**	**Ref**	**0.083**
Married	1.850	1.598	0.626–4.081	
Divorced	4.100	5.347	0.906–3.572	
Widowed	4.484	3.848	1.233–12.011	
Separated	1.708	1.541	0.741–3.203	
Types of family				
Nuclear	1	**1**	**Ref**	**0.247**
Joint	1.884	1.541	0.741–3.203	
Education level				
Illiterate	1	**1**	**Ref**	**0.128**
Basic education	2.574	2.499	0.926–6.744	
Secondary and above	1.055	1.659	0.560–4.918	
Occupation				
Unemployed	1	**1**	**Ref**	**0.088**
Employed	1.619	1.625	0.930–2.854	
Current alcohol use				
No	1	**1**	**Ref**	**0.299**
Yes	1.973	1.575	0.668–3.711	
High risk behavior				
No	1	**1**	**Ref**	**0.447**
Yes	2.022	1.260	0.694–2.290	
Spouse of migrant labor worker				
No	1	**1**	**Ref**	**0.776**
Yes	1.751	0.950	0.454–1.804	
CD4 value				
< 100 (cells/mm^3^)	1.831	1.128	0.487–2.613	0.004*
100–200 (cells/mm^3^)	0.210	0.173	0.047–1.634	
> 200 (cells/mm^3^)	1	**1**	**Ref**	
Transportation cost				
No	1	**1**	**Ref**	**0.282**
Yes	2.245	1.420	0.749–2.691	
Health worker behave				
Very friendly	1.182	1.674	0.550–5.093	0.007*
Friendly	0.929	0.965	0.384–2.425	
Indifferent	3.882	3.875	1.266–11.863	
Unfriendly	1	**1**	**Ref**	

* Statistically significant; References are in bold

## Discussion

WHOQoL-HIV BREF is a sensitive and reliable tool developed by WHO for researchers to assess the subjective well-being and HRQoL of PLHIV across diverse cultures and populations. To ensure relevance across diverse social and cultural context, it has been validated in multiple languages, including Nepali [26–28].

The WHOQoL-HIV BREF includes physical, psychological, social, environmental, level of independence, and spirituality domains. Assessment across these domains help determine the overall quality of life of PLHIV [29]. With effective active ART, life expectancy has increased; however, PLHIV continue to experience co-morbidities. Additionally, the absence of curative treatment means individuals continue to bear the burden of HIV/AIDS, and assessing QoL in this population remains important. In our study, assessment across these domains indicated a generally poor quality of life among PLHIV, which was consistent with findings from a study in Indonesia but differed from results reported in Ethiopia [24, 30]. Differences in QoL scores across studies may reflect variations in social and cultural factors, including stigma and discrimination.

Among the six domains investigated, on a 100-point scale, the environmental domain had the highest mean score, followed by psychological health, social relationships, level of independence, physical health, and spiritual belief. Higher mean scores indicate better quality of life. In this context, the environmental domain showed a highest score, while the spiritual domain had the lowest. Awareness programs targeting PLHIV have been supported by various organizations at the community level. Community and stakeholder engagement is considered a crucial component of ethical research and program implementation to achieve broad population coverage [31, 32]. Addressing persistent HIV-related disparities requires community-centred approaches, which play an important role in reducing inequities. Community engagement and improved understanding of the disease, vulnerabilities faced by patients and its treatment may contribute to a safer and more supportive environment for PLHIV, as supported by the wider engagement literature including studies from Jordan, India, Southeast Asia, and Thailand [33–36]. Encouraging enrollment in health insurance schemes and fostering peer-support groups may improve adherence to treatment of comorbid conditions and help reduce stigma, aligning with evidence [37].

A study from China reported findings that differed from our study, with higher scores observed in the physical domain [38]. In developed countries, the quality of health services may be higher due to advanced technologies, which may contribute to better quality of life in the physical domain compared to social and environmental domains [39, 40]. Spiritual health among PLHIV is an important aspect of overall well-being. Recognizing the significance of spirituality in the context of HIV is crucial for providing comprehensive and effective care [41, 42]. In our study, the mean score in the spiritual domain was 10.91 ± 2.33 among participants with comorbidities, which was lower than several other quality-of-life domains. Spiritual well-being may contribute positively to the mental and emotional resilience among PLHIV, supporting coping with the disease related challenges. A study conducted in the north-west region of Cameroon, reported the highest mean score in the spiritual belief domain, followed by levels of independence, physical, psychological, social, and environmental domains [43].

Comorbidity is associated with worse health outcomes, requiring more complex clinical management and potentially increasing healthcare costs, which may affect quality of life. PLHIV are at risk of developing chronic complications and comorbidities, such as non-communicable diseases and mental health disorders. The burden of comorbidities among PLHIV may be influenced by varying degrees of immune suppression, limited access to health services, and poor adherence to antiretroviral therapy [44–46]. Across the globe, different patterns of co-morbidities are observed among PLHIV. Tuberculosis, and diabetes have been reported as common comorbid conditions in different settings [47–49]. In our study, hypertension, diabetes, tuberculosis, and Hepatitis B were identified as common comorbidities. Most comorbid conditions were related to non-communicable diseases, reflecting an epidemiological transition from infectious to non-communicable diseases [50]. Moreover, Nepal is considered as a high-risk country for tuberculosis and HIV, as PLHIV have reduced immunity, making them more susceptible to tuberculosis, which may affect quality of life [11]. In some countries such as Ghana, studies have reported a high prevalence of comorbid conditions, including respiratory conditions, anemia, hypertension, cardiovascular disease and neurological conditions among PLHIV [51]. Our study found an association between comorbidity status and quality of life, consistent with studies conducted in Jordan [51], north-east Ethiopia [12]. These studies also reported a higher prevalence of diabetes mellitus and hypertension among individuals with comorbidities.

The distribution of comorbid conditions may vary across countries and settings, reflecting differences in epidemiological and contextual factors, including health literacy [52].

In our study, marital status was found to have an association with co-morbidities in bivariable analysis. Specifically, individuals who lived separately from their partners were more likely to report comorbid conditions. These findings are consistent with studies conducted in Ghana [8, 47]. This pattern may be related to reduced social or familial support among individuals living alone [53].

Low CD4 count is linked to neurocognitive disorders [49]. CD4 count (aOR 1.128, CI 0.487–2.613) was found associated with co-morbidities in our study, consistent with the findings from studies conducted in Thailand [54] and South Brazil [55]. Alcohol misuse may contribute to the progression of HIV/AIDS and complicate treatment, including increasing the risk of liver disease [56]. As a recognized risk behavior, alcohol use may affect adherence to complex medication regimens and may be associated with comorbid conditions, as reported in studies from Thailand [54], Ghana [49] and South Brazil [55].

The findings of this study have several important implications. Strengthening routine screening, promoting patient-centred care, enhancing health education, and fostering community engagement are important strategies for improving overall well-being. Additionally, addressing psychosocial and spiritual needs such as by accommodating community members’ views in designing interventions may support more comprehensive care [56, 57].

## Conclusions

There was a notable presence of comorbidities among PLHIV. The presence of these comorbidities was associated with various factors, including marital status, family structure, educational attainment, occupation, current alcohol consumption, having a spouse engaged in migrant labour work, CD4 count, transportation expenses, perceived behaviour of healthcare workers, and overall quality of life across multiple domains, except for the physical domain. The study findings indicate that the quality of life among individuals living with HIV was generally poor.

- The prevalence of comorbidities was higher among people living with HIV; regular health check-ups may be beneficial. Incorporating quality-of-life assessments into routine clinical practice may help identify individuals with comorbid conditions and reduced well-being, thereby facilitating timely and targeted interventions.
- Patients recently enrolled in antiretroviral therapy (ART) may require additional attention and appropriate counselling, as they reported lower quality-of-life scores and may have limited awareness or adaptation to their health condition.
- Healthcare providers can adopt a supportive and empathetic approach, provide comprehensive information regarding treatment duration and management, and ensure that quality-of-life considerations are integrated into patient care.
- Integrated interventions that address both health and social factors, including community engagement strategies are critical.

### Strengths and limitations

Among the limited number of studies conducted in Nepal, this research contributes to the evidence base by examining the occurrence of comorbidities, health-related quality of life, and associated factors among PLHIV. The cross-sectional design of this study limits causal interpretation; therefore, the identified factors should be interpreted as associations. Although laboratory reports were verified by a consultant physician, the absence of a standardized definition and uniform assessment of comorbidity remains a limitation. Some conditions, including HBV and HCV, may not have been consistently confirmed through diagnostic testing, which could affect the accuracy of comorbidity estimates.

## Supplementary Information


Supplementary Material 1. Descriptive statistics of the WHO HIV-BREF Score of HIV patients.


## Data Availability

The datasets used and/or analysed during the current study are available from the corresponding author upon reasonable request.

## Abbreviations

AIDS: Acquired immunodeficiency syndrome
aOR: Adjusted Odds Ratio
ART: Antiretroviral Therapy
CVD: Cardiovascular Diseases
HIV: Human immunodeficiency virus
HRQoL: Health-related quality of life
PLHIV: People Living with HIV
TB: Tuberculosis
WRH: Western Regional Hospital
