# Supplementary material for: Comorbidities, health-related quality of life and its associated factors among people living with HIV/AIDS in Gandaki Province of Nepal
Source: BMC Public Health. 2026 Jun 20;26:2494. doi: 10.1186/s12889-026-28192-5 (PMC13528008; doi:10.1186/s12889-026-28192-5)
Supplement: Supplementary file 1 [file 12889_2026_28192_MOESM1_ESM.docx]

Supplementary File 1: Descriptive Statistics of the WHO HIV-BREF Score of HIV Patients

| **Domains** | **Minimum** | **Maximum** | **Mean** | **Stand. Deviation** |
| --- | --- | --- | --- | --- |
| Physical Health | 5 | 17 | 11.17 | 2.14 |
| Psychological Health | 8 | 23 | 15.88 | 2.26 |
| Level of Independence | 4 | 17 | 11.38 | 2.24 |
| Social Relations | 5 | 20 | 12.08 | 2.39 |
| Environmental Health | 12 | 39 | 25.23 | 4.06 |
| Spirituality Belief | 5 | 16 | 10.35 | 2.26 |
| Overall QoL | 2 | 10 | 6.29 | 1.87 |
